# Environmental filters drive functional similarity in disjunct ferruginous outcrops of Eastern Amazonia

**DOI:** 10.3389/fpls.2025.1695218

**Published:** 2026-01-28

**Authors:** Viviane Vasconcelos Chaves, Priscila Sarmento, Arianne Flexa de Castro, José Tasso Felix Guimarães, André Luiz de Rezende Cardoso, Lourival Tyski, Rayara do Socorro Souza da Silva, Thyago Gonçalves Miranda, Sílvio Ramos, Cecílio Caldeira, Markus Gastauer

**Affiliations:** 1 Instituto Tecnológico Vale, Belém, Pará, Brazil; 2 Universidade Federal de Viçosa, Viçosa, Minas Gerais, Brazil; 3 Vale S.A, Belém, Brazil; 4 ISI-™, Belém, Brazil

**Keywords:** Araguaia, Carajás, conservation, functional similarity, functional traits

## Abstract

Ferruginous outcrops are ecologically formations that host high biodiversity and edaphic endemism. While *canga* outcrops in Carajás have been the focus of more extensive research, ferruginous outcrops in the Araguaia remain poorly studied, especially with respect to their functional ecology and conservation value. We evaluated the soils, floristic and functional compositions of plant communities on ferruginous outcrops in Carajás and the Araguaia, with the goals of comparing edaphic conditions, floristic compositions, and functional strategies between these disjunct regions and identifying patterns relevant for biodiversity conservation. A total of 129 plots were sampled spanning grassland (GS), shrubland (SB), and woodland (WD) formations. In all plots, soil samples were collected, and plant traits related to resource acquisition (SLA, leaf N, N:P), reproductive strategies (fruit dimensions), and interaction modes (dispersal and pollination syndromes) were measured. Herbaceous and woody communities (trees and treelets with dbh ≥= 3 cm) were analyzed separately. Functional similarity was assessed via community-weighted means and multivariate trait space analyses. Despite exhibiting moderate floristic similarity between regions and edaphic differences, both regions share acidic soils with low phosphorus (P) availability, a condition that imposes similar constraints on resource acquisition. Open formations (GS, SB) in both regions showed functional convergence, indicating similar environmental filters. In contrast, woody communities, especially those in WD, presented pronounced differences in trait composition, reflecting differences in local conditions and ecological history. This study highlights the complementary conservation value of ferruginous outcrops in Carajás and the Araguaia. The functional similarities in open formations suggest that these environments may exhibit ecological strategies associated with similar environmental conditions. Recognizing and protecting these unique environments is essential to ensure their long-term ecological resilience.

## Highlights

- Soil, floristic and functional compositions of different vegetation formations in ferruginous outcrops from the Araguaia were compared with those from the Carajás region.
- Despite different climatic and geological contexts, the vegetation formations in both regions exhibit similar gradients of environmental severity.
- Considerable species overlap between regions highlights the role of historical connections and ecological corridors between regions.
- Environmental filters that structure open formations select functionally similar species in both regions.
- Floristic differences between regions require conservation efforts to preserve the biological resources of the Araguaia for future generations.

## 1 Introduction

Ferruginous outcrop ecosystems, often characterized by highly diverse rupestrian vegetation, are disjunct formations from different regions of the world and are generally characterized by significant environmental heterogeneity, resulting in mosaics of plant communities with high endemism (Jacobi et al., 2007; Gibson et al., 2010). In Brazil, an emblematic example is found in the Carajás Massif, Eastern Amazonia, where ferruginous outcrops, locally known as *cangas*, harbor hundreds of herbaceous and shrub species (Mota et al., 2015, 2018), 38 of which are considered edaphic endemics (Giulietti et al., 2019). While Carajás *cangas* has received scientific attention because of their exposure and mineral potential (Gastauer et al., 2021; Giulietti et al., 2019), they are not the only iron-rich substrates in the Amazon that support such vegetation. Ferruginous outcrops from the Lower Araguaia River basin, located in southeastern Pará, are described as ferricretes and occur under tropical geoenvironmental conditions that favor the formation of iron-rich duricruts (Da Costa, 1991). Although they differ geologically from the Carajás outcrops, these formations support similar vegetation formations (Reis et al., 2025). However, the community structure and functional attributes of Araguaia ferruginous outcrops have not yet been systematically investigated. Previous studies have addressed floristic composition and phylogenetic structure across rupestrian and ferruginous landscapes (Zappi et al., 2017; Neves et al., 2018; Andrino et al., 2020; Massante et al., 2023), but a clear understanding of how environmental and evolutionary filters shape plant functional traits in these disjunct systems from the southeastern Amazon remains lacking.

Assessing the extent to which these disjunct ferruginous systems share ecological properties is crucial for understanding how plant communities assemble on iron-rich substrates and respond to long-term environmental pressures (Neves et al., 2018; Massante et al., 2023). While these ecosystems are currently fragmented, they may represent remnants of a broader floristic and functional continuum (Silveira et al., 2016). This perspective raises the possibility that, under drier climatic conditions in the past, and potentially under future climate change scenarios (Hopper, 2009), these systems may have been, or could once again become, ecologically connected. Functional comparisons among local floras are crucial for revealing whether similar selective pressures lead to convergent trait syndromes or whether historical contingencies and isolation have resulted in distinct functional strategies (Fu et al., 2014). Such analyses can shed light on past biogeographical connections among ferruginous landscapes and inform conservation planning by identifying functionally complementary or irreplaceable assemblages. In a rapidly changing world, understanding functional differentiation also offers a practical basis for anticipating ecosystem responses to disturbances. It further supports the design of restoration and translocation efforts that maintain ecological functionality and adaptive potential (Lavorel and Garnier, 2002; Díaz et al., 2007; Funk et al., 2017). This knowledge gap is particularly concerning because ferruginous outcrops are located in regions heavily impacted by land-use changes. Both areas are located within the so-called “Arc of Deforestation”, a zone that has undergone rapid transformations since the 1980s due to pasture expansion, mechanized agriculture, mining, and infrastructure development, resulting in intense habitat degradation (Skirycz et al., 2014; Schaefer et al., 2016; Barbosa et al., 2020; Marques et al., 2020; Martins et al., 2021). These circumstances highlight the urgency of understanding the floristic and functional patterns of these ferruginous ecosystems to inform more effective conservation strategies and management plans. Evaluating their degree of functional similarity could thus reveal underlying assembly processes and inform conservation strategies aimed at preserving resilient ecosystems.

Traits such as leaf morphology, reproductive mode, dispersal capacity, and pollination syndromes are closely associated with ecological performance and allow us to infer reveal how species respond to environmental filters or local biotic interactions (Dellinger, 2020; Zanetti et al., 2020; Rosas-Guerrero et al., 2014). A comparison of ferruginous outcrops from Carajás and the Araguaia can reveal whether communities share similar functional strategies or whether regional divergence in trait distributions reflects distinct environmental or historical pressures. Through these trait-environment relationships, we can better understand the mechanisms of community assembly and the environmental filters that structure these ecosystems (Díaz et al., 2022; Da Cruz et al., 2021). Moreover, functional traits are directly associated with ecosystem functioning and the provision of ecosystem services, providing a robust basis to guide conservation and restoration actions (Laughlin et al., 2018; Madani et al., 2018).

In this context, this study aimed to evaluate soil, floristic composition, taxonomic diversity, and functional similarity between plant communities growing on iron duricrusts from the Carajás Massif and those from the Lower Araguaia Basin, Eastern Amazonia, Brazil. Considering the structural gradient characteristics of these ecosystems, we analyzed three representative vegetation formations (grassland, shrubland, and woodland). We expect that (1) the soil properties are similar, indicating the dominance of similar ecological filters despite their different geological origins. (2) Ferruginous outcrops from Araguaia and Carajás exhibit floristic and functional similarity, particularly in open formations, reflecting potential historical connectivity and/or convergent adaptation to similar environmental constraints. (3) Taxonomic diversity will differ between regions given their distinct biogeographical contexts, which may influence species pools and assembly processes. To test these hypotheses, comparisons between regions were performed via Student’s t test, whereas comparisons among formations within each region were evaluated via ANOVA followed by Tukey’s *post hoc* test. Floristic composition was then compared between regions via PERMANOVA, which was supported by nonmetric multidimensional scaling (NMDS). Taxonomic diversity was assessed through rarefaction curves, and functional similarity was evaluated via community-weighted means (CWMs, tested with Wilcoxon tests), and the functional space was generated via principal coordinate analysis (PCoA).

## 2 Materials and methods

### 2.1 Study site

This study was conducted on ferruginous outcrops in two regions of southeastern Pará, Brazil: (i) the Lower Araguaia River basin and (ii) the Carajás region, which includes the Carajás National Forest and the Campos Ferruginosos National Park. Ferruginous outcrops in southeastern Amazonia correspond to substrates of iron duricrusts formed through prolonged tropical weathering processes (lateritization), involving repeated cycles of iron dissolution and reprecipitation, and the cementation of lateritic residuum or colluvial materials by Fe(III) oxyhydroxides (mainly goethite and hematite), with variable silica and Mn oxide contents (Monteiro et al., 2018; Da Costa, 1991; Da Silva et al., 2024).

In the Araguaia, ferricretes predominantly result from the cementation of colluvial mantles derived from preexisting iron duricrusts, and recent studies also indicate the influence of ultramafic and ophiolitic units in the region, which hosts Mg-, Ni- and Cr-rich lithotypes (Barros and De Sousa Gorayeb, 2019; Da Costa et al., 2022). These crusts, which are generally thin to moderately thick and matrix rich, overlie metasedimentary rocks of the Couto Magalhães Formation and occur on low-relief surfaces (~56–200 m asl). Their development is associated with a relatively younger landscape shaped by reworked and recemented materials rather than thick, *in situ* weathering profiles (Sahoo et al., 2025; Reis et al., 2025; Da Costa, 1991).

In contrast, in the Carajás Mountains, iron duricrusts (or canga) overlie ancient and polycyclic laterites that initially formed *in situ* over Archean banded iron formations (BIFs), which host extensive high-grade iron ore deposits (Nunes et al., 2015). These iron duricrusts have also undergone episodes of reworking and recementation, forming ferricretes characterized by clast-supported breccias composed of hematite, BIF fragments, and other lithotypes. These breccias typically cap high plateaus and ridges at elevations of approximately 850 m. Their high degree of induration, structural control, and discontinuous distribution suggest a strong association with an older and more mature landform shaped by long-term supergene processes (Da Silva et al., 2024; Sahoo et al., 2025; Reis et al., 2025; Martins et al., 2021; Monteiro et al., 2018; Spier et al., 2019).

Iron-cemented colluvial deposits, here classified as ferricretes, differ in parent material (colluvial ferricretes over metasediments in the Araguaia *vs*. in-place ferricretes over BIF in the Carajás Mountains), relative age/maturity (younger formations in the Araguaia), altitudinal setting, mineralogical composition (more SiO2 in the Araguaia, Guimarães et al. In Prep.), and duricrust architecture (matrix-rich *vs*. breccia/pavement), they support similar savanna-like vegetation types in both the Carajás Mountains and the Araguaia. Triggered by contrasting soil depths, porosity/fissure density, hydrological regime (perched water versus lateral flow), and nutrient status (notably low available P with variable base cations), small-scale mosaics of grasslands (GS), shrublands (SB) and woodlands (WD) emerge in both regions. GS occur on shallow, acidic, nutrient-poor, seasonally flooded soils dominated by Poaceae, Cyperaceae, Asteraceae, and Xyridaceae (Mota et al., 2018; Schaefer et al., 2016; Monteiro et al., 2025), whereas SB occur where soils accumulate in fissures of ferruginous outcrops. WD are located on deeper, more nutrient-retentive soils at the margins of ferruginous outcrops; in Carajás, these WD are also common in soils that accumulate small depressions, creating forest islands surrounded by naturally open vegetation (Mitre et al., 2018). In these environments, common species include *Xylopia aromatica* (Annonaceae), *Licania* sp. (Crysobalanaceae), *Dipteryx odorata* (Fabaceae), *Parkia platycephala* (Fabaceae), and *Tapira guianensis* (Anacardiaceae) (Monteiro et al., 2023).

**Figure 1:**
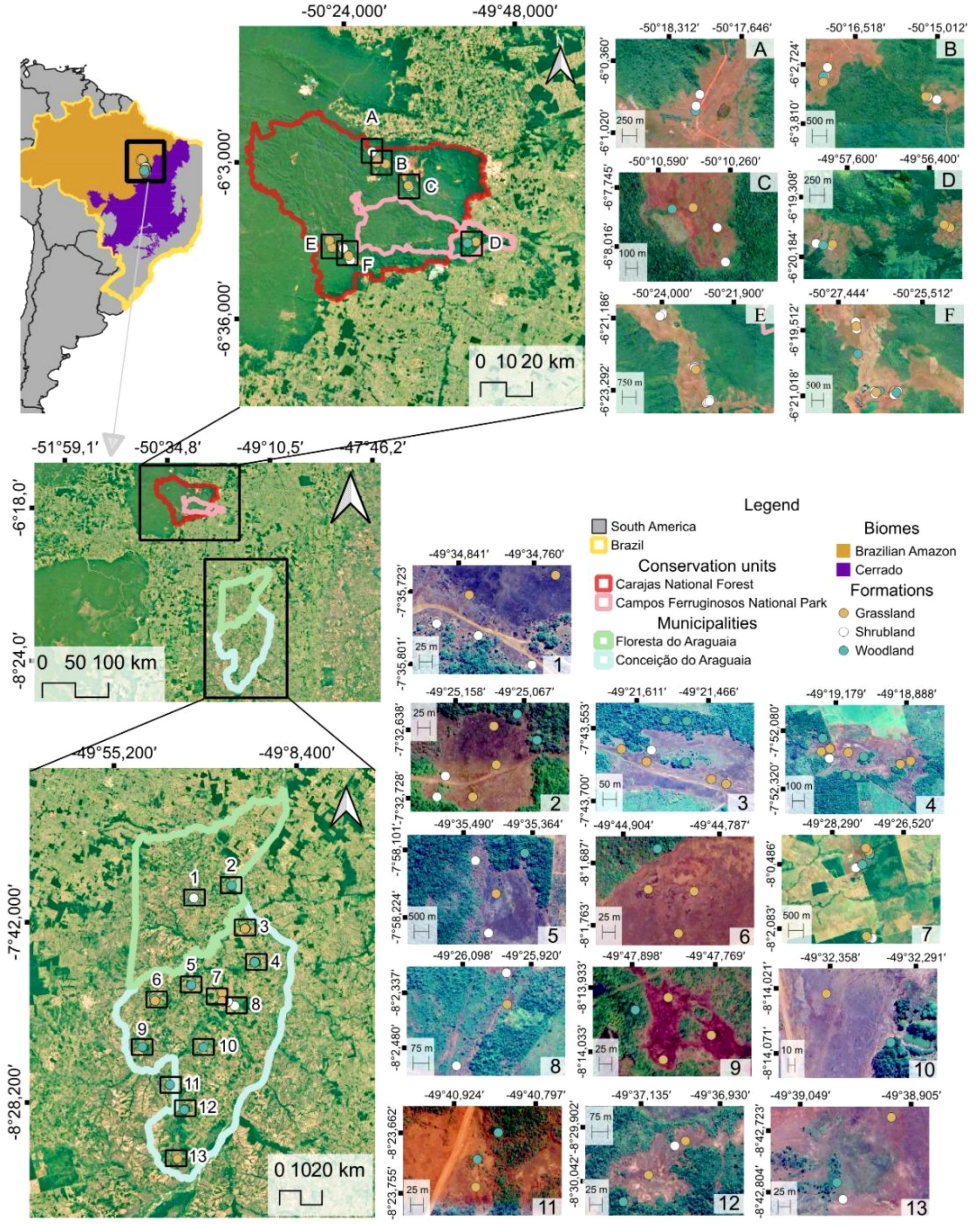
Locations of the study sites and sampling points in the ferruginous outcrops of the Carajás region **(A–F)** and the Lower Araguaia River basin (1–13).

The sampling in the Lower Araguaia River basin spans the municipalities Floresta do Araguaia and Conceição do Araguaia (**Figure 1**). This area lies within the transition zone between the Amazon and Brazilian savanna (Cerrado) biomes, forming an ecotone where features of both biomes coexist (Garcia et al., 2017; De Oliveira et al., 2020). Like other ecotonal areas, the region harbors high biodiversity (Dagosta and Pinna, 2017). The climate is tropical monsoon (Am, according to Köppen’s classification) (Alvares et al., 2013), with an annual precipitation of approximately 2,000 mm. The rainy season occurs from November to May, whereas the dry season extends from June to October (Hoffmann et al., 2018). Carajás *cangas* were sampled in the Carajás National Forest (a class IV conservation unit created in 1998) and the Campos Ferruginosos National Park (a class II conservation unit created in 2017) (Silva et al., 2024; INSTITUTO CHICO MENDES DE CONSERVAÇÃO DA BIODIVERSIDADE – ICMBio, 2024), (**Figure 1**). Together, these areas form part of a mosaic of conservation initiatives that cover ~12,000 km^2^, which is important for preserving Amazonian biodiversity (Giannini et al., 2025). The climate is tropical savanna (Aw, Köppen’s classification), with a dry period between May and September and peak rainfall from January to March. The annual precipitation is approximately 2,300 mm, with ~80% falling in the rainy season (Alvares et al., 2013). Natural vegetation is dominated by a matrix of dense or open evergreen submontane forests above latossols, in which ironstone outcrops are inserted (Giulietti et al., 2019). The main threats to these ecosystems are mining, followed, especially along the borders of the conservation units, by more or less severe fire events and the dispersion of invasive species.

### 2.2 Field surveys

The vegetation surveys were conducted in 20 × 10 m permanent plots. Plots were installed in areas visibly free of disturbance, i.e., absence of recent fire signs, trails, invasive species, or selective logging activities, and spaced a minimum of 50 m apart to encompass the environmental heterogeneity of each ferruginous outcrop patch. The plots were georeferenced and, when possible, marked with 50 cm PVC tubes. In each plot, all trees and shrubs with a circumference at a height greater than 10 cm were considered part of the woody stratum and were tagged and identified to the species level. To assess the herbaceous stratum, five 1 × 1 m subplots were established within each 20 × 10 m plot, where all the species were identified and their relative cover was estimated in the field.

A total of 72 plots (1.44 ha) were sampled in the Araguaia; these plots were distributed among the GS (33 plots, 0.66 ha), SB (19 plots, 0.38 ha), and WD (20 plots, 0.4 ha) formations. For comparison, we used a vegetation database from the Carajás region comprising 48 plots (0.96 ha) distributed among the GS (11 plots, 0.22 ha), SB (29 plots, 0.48 ha), and WD (8 plots, 0.16 ha) formations (Gastauer et al., 2020). The number of plots differs between regions and specific formations as a function of patch size, accessibility, and dominance of each vegetation type in each region.

To detect soil fertility and granulometry, we collected composite samples of approximately 600 g in all 10 × 20 m plots. Sampling was carried out at five points: at each end of the x- and y-axes and at the center. The samples were stored in plastic bags and transported to the laboratory for analysis. Granulometry and fertility were analyzed following the methods described by Embrapa (Teixeira et al., 2017).

### 2.3 Collection and analysis of functional traits

To compare the functional traits of plant communities in ferruginous outcrops in the Araguaia and in the Carajás region, we selected key functional traits associated with resource use strategies, reproduction, and dispersal (**Table 1**).

**Table 1:** Functional traits addressed in this study and their biological significance.

Traits	Unit	Acquisition	Ecological significance
Plant height	m	Field measurements using a measuring tape for shrubs and a clinometer for tree	Important for competition and light acquisition, especially in woody communities (Perez-Harguindeguy et al., 2013).
Leaf length (LL)	cm	Caliper measurements of field-collected leaves	Related to light capture efficiency and resource allocation (Cornelissen et al., 2003).
Leaf width (LW)	cm	Caliper measurements of field-collected leaves	Represents a response to resource availability, and to the leaf’s water and energy balance (Perez-Harguindeguy et al., 2013).
Specific leaf area (SLA)	cm²/g	Field collected leafs were scanned (HP), dried at 60 °C and weighted with a precision balance (Model); leaf area was detected from the scans using imageJ	Indicates the balance between rapid resource acquisition and conservative strategies (Wright et al., 2004; Perez-Harguindeguy et al., 2013).
Leaf nitrogen content	g/kg	Analyzed in the laboratory using plasma atomic emission spectrometry (ICP–AES)	Associated with photosynthetic capacity and growth rate (Wright et al., 2004).
Leaf N:P ratio	–	Analyzed in the laboratory using plasma atomic emission spectrometry (ICP–AES)	Reflects nutrient use efficiency and indicates whether biomass production is limited by nitrogen or phosphorus (Güsewell, 2004).
Fruit length (FL)	cm	Caliper measurements of field collected fruits, consultations of specimens from the Herbarium of the, Carajás (HCJS), and the Herbarium of the Museu Paraense Emílio Goeldi (MPEG), complemented with records from SpeciesLink and Flora e Funga do Brasil.	Indicator of reproductive investment, influencing dispersal mechanisms and seed survival (Bentos et al., 2014; Páz-Dyderska and Jagodzinki, 2024).
Fruit width (FW)	cm	Caliper measurements of field collected fruits, consultations of specimens from the Herbarium of the, Carajás (HCJS), and the Herbarium of the Museu Paraense Emílio Goeldi (MPEG), complemented with records from SpeciesLink and Flora e Funga do Brasil.	Influences dispersal mechanisms and reflects the reproductive strategies of plants (Bentos et al., 2014; Páz-Dyderska and Jagodzinki, 2024).
Dispersal syndrome	–	Obtained from the literature (Kuhlmann and Ribeiro, 2016; Jacobi and Do Carmo, 2011; Dutra et al., 2009).	Different ecological strategies related to fruit and seed dispersal, as well as seedling survival (Galetti et al., 2013; Pereira et al., 2022).
Pollination syndrome	–	Obtained from the literature (Kuhlmann and Ribeiro, 2016; Jacobi and Do Carmo, 2011; Dutra et al., 2009).	Represents the set of floral traits associated with the main pollination agents of a species (Fontaine et al., 2006; Stang et al., 2007; Pinto et al., 2020; Pereira et al., 2022).

The samples included all the species that represented approximately 80% of the individuals in the woody community and 75% of the total vegetation cover in the herbaceous community. For each species that fulfills these criteria, we selected five visually healthy adult individuals per species. Plant height was measured via a tape measure (herbs and small shrubs) and a clinometer (larger shrubs and trees), respectively. For leaf trait analyses (LL, LW, SLA, N, N:P), three or more sun-exposed leaves from these individuals were collected. The leaf carbon content was not measured; therefore, the leaf nitrogen content was used as a proxy for the leaf carbon-to-nitrogen ratio, as variations in this ratio are mainly determined by the nitrogen content (Xiong et al., 2022; Xu et al., 2020).

For species not fruiting during the field collections, we consulted the collections of the Carajás (HCJS) and the Museu Paraense Emílio Goeldi (MPEG). Both MPEG collections house most species from Carajás’s *cangas*, and four years of intense sampling in the Araguaia were deposited in the HCJS collection. For field and herbarium measurements, we sampled between one and five fruits from at least five individuals. For species not available in sufficient number at both institutions, we checked the SpeciesLink system (CRIA (Centro de Referência e Informação Ambiental), 2017) and the Flora and Fungi of Brazil (REFLORA, 2025). Whenever possible, we prioritized individuals collected from ferruginous environments or regions with similar ecological characteristics (e.g., same biome or geographic zone) to minimize bias associated with environmental plasticity. For analysis, the categorical variables dispersal and pollination syndromes were converted into binary traits.

### 2.4 Statistical analyses

To evaluate whether the soil fertility of the ferruginous outcrops from Carajás and the Araguaia is similar, we compared the physicochemical soil properties between regions. To compare the same vegetation formation across regions, we applied Student’s t test, and to compare formations within each region, we used one-way ANOVA followed by Tukey’s *post hoc* test. The analyzed variables included pH, Mehlich-1 extractable phosphorus (Pmeh), potassium (K), sodium (Na), nitrogen (N), sulfur (S), calcium (Ca), magnesium (Mg), aluminum (Al), organic matter (OM), copper (Cu), iron (Fe), manganese (Mn), zinc (Zn), sum of bases (SB), and clay content.

To understand the similarities in floristic composition between the ferruginous outcrops, we checked whether the species detected within the surveys conducted in the Araguaia also occur in the ferruginous outcrops of Carajás (Mota et al., 2018). For that, we considered only species identified to the species level. Next, differences in community composition were assessed via non-metric multidimensional scaling (NMDS) based on Bray-Curtis distances, implemented through the function ‘metaMDS’ from the ‘vegan’ package (Oksanen et al., 2024). The significance of differences was tested via permutational multivariate analysis of variance (PERMANOVA) via the ‘adonis’ function. To assess whether species richness and diversity differed between ferruginous formations in the two regions, we constructed rarefaction curves on the basis of the number of sampled individuals (woody community) and vegetation cover (herbaceous community). This approach enables the estimation and comparison of diversity while controlling for differences in sample size. Rarefaction and subsequent extrapolation were conducted with the ‘iNEXT’ package (Chao et al., 2014).

To evaluate the functional equivalence between the plant communities of the ferruginous outcrops from Carajás and the Araguaia, we calculated the community weighted means (CWMs) for all functional traits via the ‘dbFD’ function from the ‘FD’ package and the Gower distance. CWMs summarize the average trait value in a community, weighted by species abundance or cover, thus reflecting the dominant functional strategies that drive ecosystem processes and responses to environmental conditions. This approach provides a robust basis for comparing functional compositions among regions and formations. The Gower distance was adopted because it allows the integration of continuous and categorical traits while tolerating missing values. Comparisons of all CWMs among vegetation types among formations (GS, SB, WD) within each region were conducted via the Kruskal-Wallis test, followed by Dunn’s *post hoc* test. Non-parametric tests were chosen because the CWM data did not meet parametric assumptions even after transformation. To compare the CWM of formations between regions, we used Wilcoxon tests.

On the basis of CWMs, we calculated the mean Bray-Curtis dissimilarities among regions for the three formations to quantify functional differentiation between plant communities. The Bray-Curtis index was chosen because it is sensitive to differences in species abundance and functional composition, providing a robust measure of the ecological distance between communities. The Bray-Curtis index ranges from 0, indicating functionally identical communities, to 1, indicating completely different samples (Legendre and Legendre, 1998). To facilitate ecological interpretation, dissimilarity values were categorized as follows: < 0.25 as low dissimilarity (high similarity), 0.25-0.50 as moderate dissimilarity, and > 0.50 as high dissimilarity (low similarity).

The functional trait space (Gastauer et al., 2020) from each formation from both regions was calculated via principal coordinate analysis (PCoA) on the basis of the Gower distance among all species considering the complete functional trait matrix. This ordination approach provides a multidimensional representation of the functional relationships among formations and regions. The analysis was performed with the ‘cmdscale’ function in the ‘vegan’ package (Oksanen et al., 2024). This approach allows the integration of traits measured at different scales, making it particularly suitable for plant functional ecology studies. Significance was tested via PERMANOVA. All the analyses were conducted in R version 4.3.2 (R Core Team, 2024).

Sample sizes differ among formations and regions, reflecting their natural occurrence and accessibility in the field. No specific resampling or weighting procedure was applied to equalize the sampling effort. However, diversity comparisons were standardized through rarefaction and extrapolation curves, and the multivariate analyses (NMDS, PERMANOVA, and PCoA) used are robust to unequal sample sizes. Non-parametric tests (Kruskal–Wallis, Dunn, and Wilcoxon) were applied to detect general trends, and the results were interpreted with caution given their potential sensitivity to unbalanced groups.

## 3 Results

In the Lower Araguaia River basin, we recorded 174 species (113 genera, 55 families), including 78 tree species (60 genera, 26 families) and 96 herbaceous species (53 genera, 27 families). In the Carajás plots, 161 species were detected (104 genera, 64 families), comprising 72 woody species (49 genera, 35 families) and 89 herbaceous species (55 genera, 29 families). Fabaceae was the most species-rich family in the woody communities from both localities. Among the herbaceous communities, Fabaceae predominated in Araguaia plots, followed by Poaceae and Cyperaceae, whereas Poaceae dominated in Carajás plots, followed by Cyperaceae.

Although some soil attributes differed significantly between regions (**Table 2**), the qualitative patterns revealed similarities in edaphic attributes among the ferruginous outcrops. Across formations in both regions, soils presented low pH values and high contents of OM. Similarly, Ca, Mg, and P maintained low levels in all the formations. Al displayed a consistent pattern across formations, with low levels in GS, intermediate levels in SB, and high levels in WD. K showed intermediate levels in the SB and WD formations in both regions. Zn was present at high levels in the SB and WD formations, varying only in the GS, where it was high in Araguaia and intermediate in Carajás. Mn was high in all formations in Araguaia, but in Carajás, it ranged from low to intermediate. Cu showed intermediate levels in the GS of Araguaia and high levels in the GS of Carajás, being consistently high in the SB and WD formations of both regions.

**Table 2:** Soil physical–chemical properties (mean ± standard deviation) across three vegetation formations (grassland, shrubland, and woodland) in ferruginous outcrops from Carajás and the Lower Araguaia River basin, Eastern Amazonia, Brazil.

Variable	Araguaia GS (mean ± SD)	Carajás GS (mean ± SD)	Araguaia SB (mean ± SD)	Carajás SB (mean ± SD)	Araguaia WD (mean ± SD)	Carajás WD (mean ± SD)
pH	5.00 ± 0.18^a^	4.89± 0.49^A^	4.77 ± 0.32^a^*	4.48 ± 0.51^B^*	4.75± 0.31^b^*	4.20 ± 0.48^B^*
P (mg/dm³)	3.08 ± 4.75	6.19 ± 5.38	2.08 ± 1.72	7.44 ± 17.04	1.13 ± 0.83*	4.64 ± 3.54*
K (mg/dm³)	22.11 ± 7.50^c^*	49.69 ± 25.90*	32.70 ± 10.85^b^*	50.29 ± 20.46*	45.17 ± 19.09^a^	47.07 ± 14.76
Na (mg/dm³)	8.21 ± 2.75*	21.95 ± 12.54*	9.43 ± 3.76*	18.87 ± 13.75*	9.05 ± 3.74*	24.03 ± 9.70*
N(g/kg)	0.29± 0.14^b^*	0.69 ± 0.26*	0.41 ± 0.20^ab*^	0.78 ± 0.35*	0.50 ± 0.15^a^*	0.67 ± 0.17*
S(mg/dm³)	3.64 ± 1.47*	24.53 ± 44.3*	9.61 ± 17.24	7.42 ± 3.36	4.75 ± 2.30	13.20 ± 10.12
Ca(cmolc/dm³)	0.28 ± 0.73	0.28 ± 0.24^B^	0.27 ± 0.35*	0.75 ± 0.85^A^*	0.70 ± 1.02*	0.08 ± 0.05^C^*
Mg(cmolc/dm³)	0.08 ± 0.05^c^*	0.14 ± 0.08*	0.15 ± 0.11^b^	0.25 ± 0.23	0.37 ± 0.42^a^*	0.10 ± 0.04*
Al(cmolc/dm³)	0.32 ± 0.15^b^	0.55 ± 0.60^B^	1.00 ± 0.64^a^	1.00 ± 0.69^B^	1.46 ± 0.80^a^	2.00 ± 0.83^A^
OM (g/kg)	3.53± 0.93^b^*	10.80± 3.45*	4.89 ± 1.61^b^*	13.23 ± 4.06*	6.52 ± 1.76^a^*	11.13 ± 2.14*
Fe(mg/dm³)	159.22 ± 97.07*	328.09 ± 178.07^B^*	157.60 ± 69.80*	443.41 ± 105.67^A^*	166.07 ± 52.51*	366.75 ± 134.82^AB^*
Mn (mg/dm³)	5.16 ± 5.09*	2.30 ± 2.27*	8.17 ± 7.90	3.07 ± 2.88	11.41± 10.53*	1.25 ± 0.85*
Cu (mg/dm³)	0.4 ± 0.23^b^*	1.11 ± 0.34*	0.63 ± 0.34^ab^*	1.35 ± 0.48*	0.9 ± 0.49^a^	1.07 ± 0.45
Zn (mg/dm³)	0.78 ± 0.29*	0.46 ± 0.19*	0.97 ± 0.32	0.90 ± 0.76	0.93 ± 0.26*	0.59 ± 0.33*
SB (cmolc/dm³)	0.45 ± 0.77*	0.63 ± 0.38^B^*	0.54 ± 0.45*	1.20 ± 1.08^A^*	1.22 ± 1.44	0.39 ± 0.11^B^
Clay	182.50 ± 52.44^b^*	304.38 ± 80.42*	248.12 ± 82.85^ab^	247.07 ± 106.12	296.67 ± 75.28^a^	322.50 ± 138.67

pH, soil pH in water; Pmeh, Mehlich-1 extractable phosphorus; K, potassium; Na, sodium; N, nitrogen; S, sulfur; Ca, calcium; Mg, magnesium; Al, aluminum; OM, organic matter; Cu, copper; Fe, iron; Mn, manganese; Zn, zinc; SB, sum of bases; Clay, clay content. Different lowercase letters indicate significant differences among formations within the Araguaia basin, and different uppercase letters indicate significant differences among formations within Carajás according to *post hoc* Tukey tests following ANOVA. “*” indicates significant differences between regions for formations according to Student’s t test.

**Figure 2:**
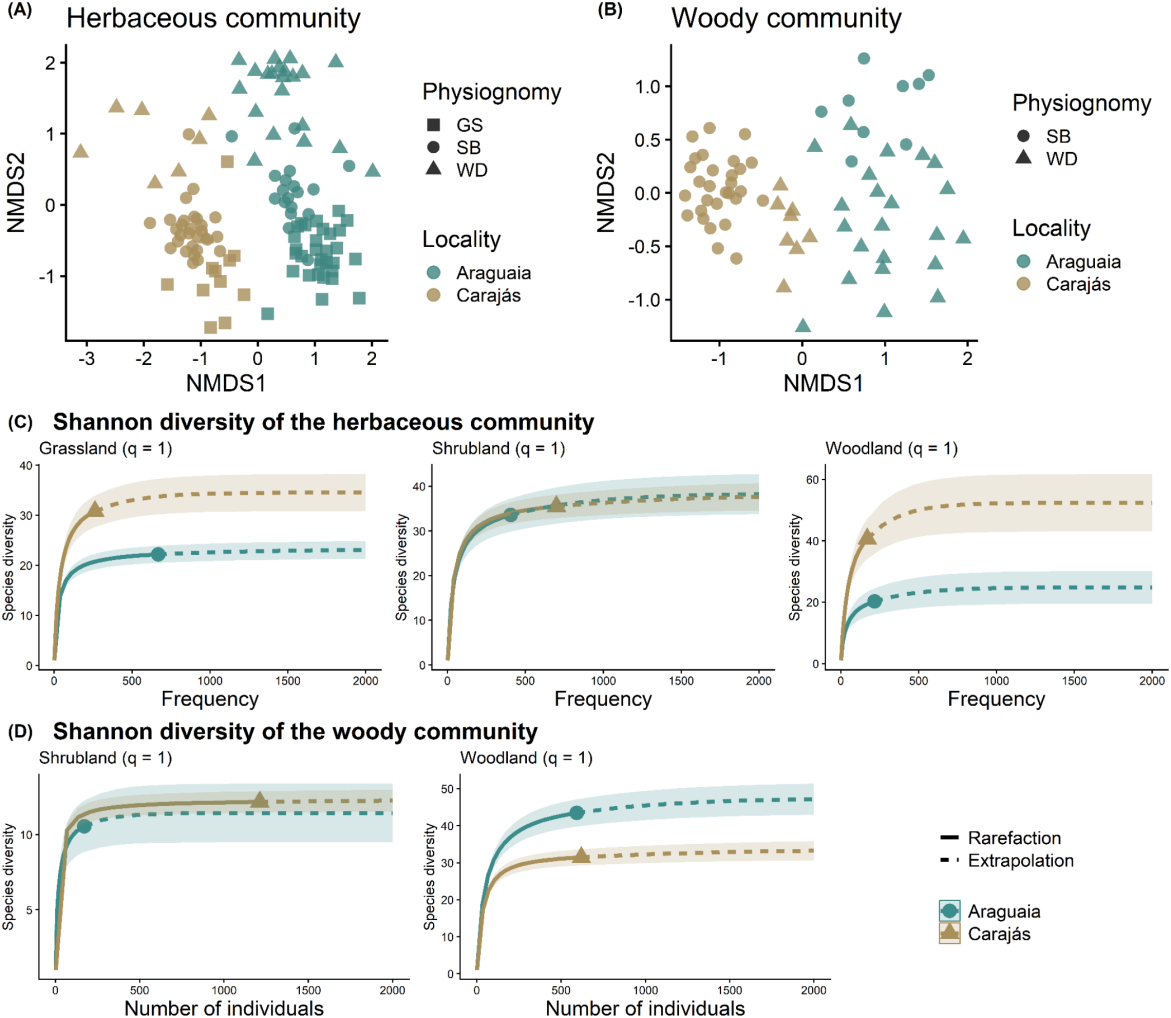
Composition and diversity of different vegetation formations from ferruginous outcrops in Carajás and the Lower Araguaia River basin. NMDS ordination based on species composition in the herbaceous **(A)** and woody **(B)** communities. Rarefaction and extrapolation curves of Shannon diversity of the herbaceous **(C)** and woody communities **(D)**. GS is Grassland, SB is Shrubland and WD is Woodland.

A total of 150 taxa were identified at the species level in the Araguaia ferruginous outcrops during this study, 83 of which (56%, **Supplementary Table S3**) were shared with Carajás. Despite this partial overlap, NMDS analysis revealed a significant difference in floristic composition between the ferruginous outcrops of Carajás and those of the Araguaia, both for the herbaceous (F = 15.00, R² = 0.40, p < 0.001; **Figure 2A**) and arboreal communities (F = 10.24, R² = 0.32, p < 0.001; **Figure 2B**). Even when analyzed separately by formation, the composition differences between the two regions remained significant (**Supplementary Figure S2**). Rarefaction and extrapolation curves revealed differences in diversity between regions, particularly in WD. In the herbaceous community, Carajás showed greater diversity in GS and WD, whereas the diversity in SB did not differ significantly between regions. In the woody community, plots from the Araguaia presented greater WD diversity than those from Carajás, whereas SB did not differ between regions (**Figures 2C, D**).

**Figure 3:**
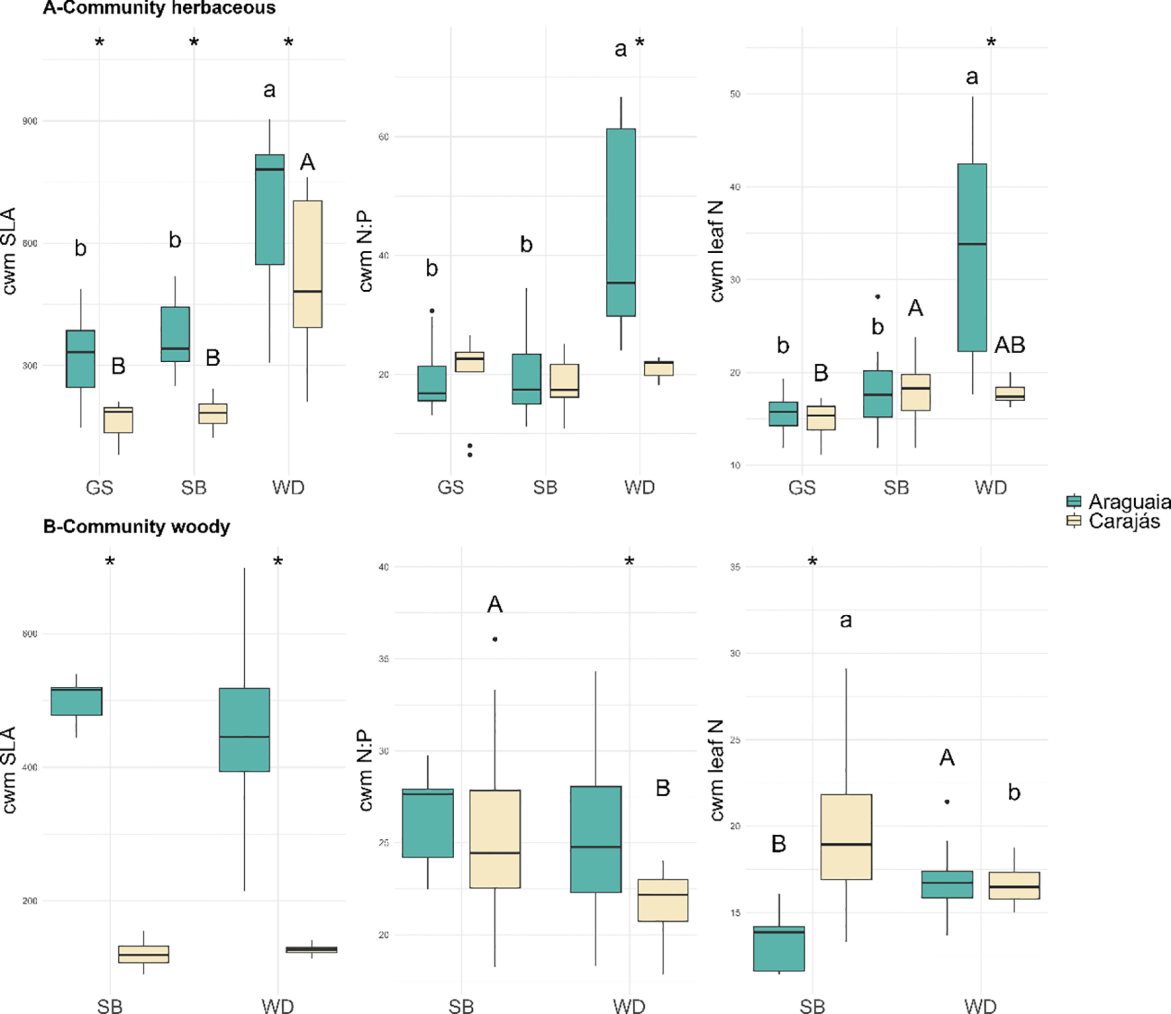
Community-weighted mean (CWM) values of species traits of different vegetation formations from ferruginous outcrops in Carajás and the Lower Araguaia River basin: **(A)** Herbaceous and **(B)** - Woody communities: (SLA, specific leaf area; N:P, leaf nitrogen-to-phosphorus ratio; N, leaf nitrogen content). Different lowercase letters indicate significant differences among Carajás formations; uppercase letters indicate differences among Araguaia basin formations; (*) indicates significant differences between localities (p < 0.05). GS is Grassland, SB is Shrubland and WD is Woodland.

CWMs of traits related to resource allocation and reproduction differed between vegetation formations but showed consistent patterns across both regions. In particular, SLA exhibited similar trends across formations, being higher in the Araguaia plots, whereas N:P and leaf N varied primarily with formation, showing fewer differences between regions. Within the herbaceous community, the N:P ratio and leaf N ratio were largely comparable between regions, with formation-specific contrasts most evident in WD (**Figure 3A**). In the woody community, height, N:P ratio and leaf N tended to be greater in the SB of Carajás; in WD, the main contrast involved higher N:P ratios in Araguaia plots, which was consistent with greater P limitation (**Figure 3B**). With respect to morphology, leaf and fruit length showed no clear between-region differences within GS or SB, whereas leaf width was greater in SB from Carajás (**Supplementary Figure S5**). Interaction-related traits showed more localized differences: anemochory was more frequent in GS and WD from the Araguaia; autochory predominated in herbaceous formations from the Araguaia and in the woody community of Carajás WD; and zoochory was prevalent across herbaceous and woody formations in Carajás (**Supplementary Figure S6**). In terms of pollination, anemophily was more common in GS from the Araguaia region (**Supplementary Figure S7**).

Functional similarity based on the CWM indicated moderate similarity between regions for the herbaceous communities of SB and GS (Bray-Curtis distances = 0.2753 ± 0.0959 and 0.3705 ± 0.1074, respectively) and greater dissimilarity in WD (0.5685 ± 0.1299). In the woody community, all the formations presented high functional dissimilarity between localities (0.5457 ± 0.0501 for SB and 0.5025 ± 0.1088 for WD).

**Figure 4:**
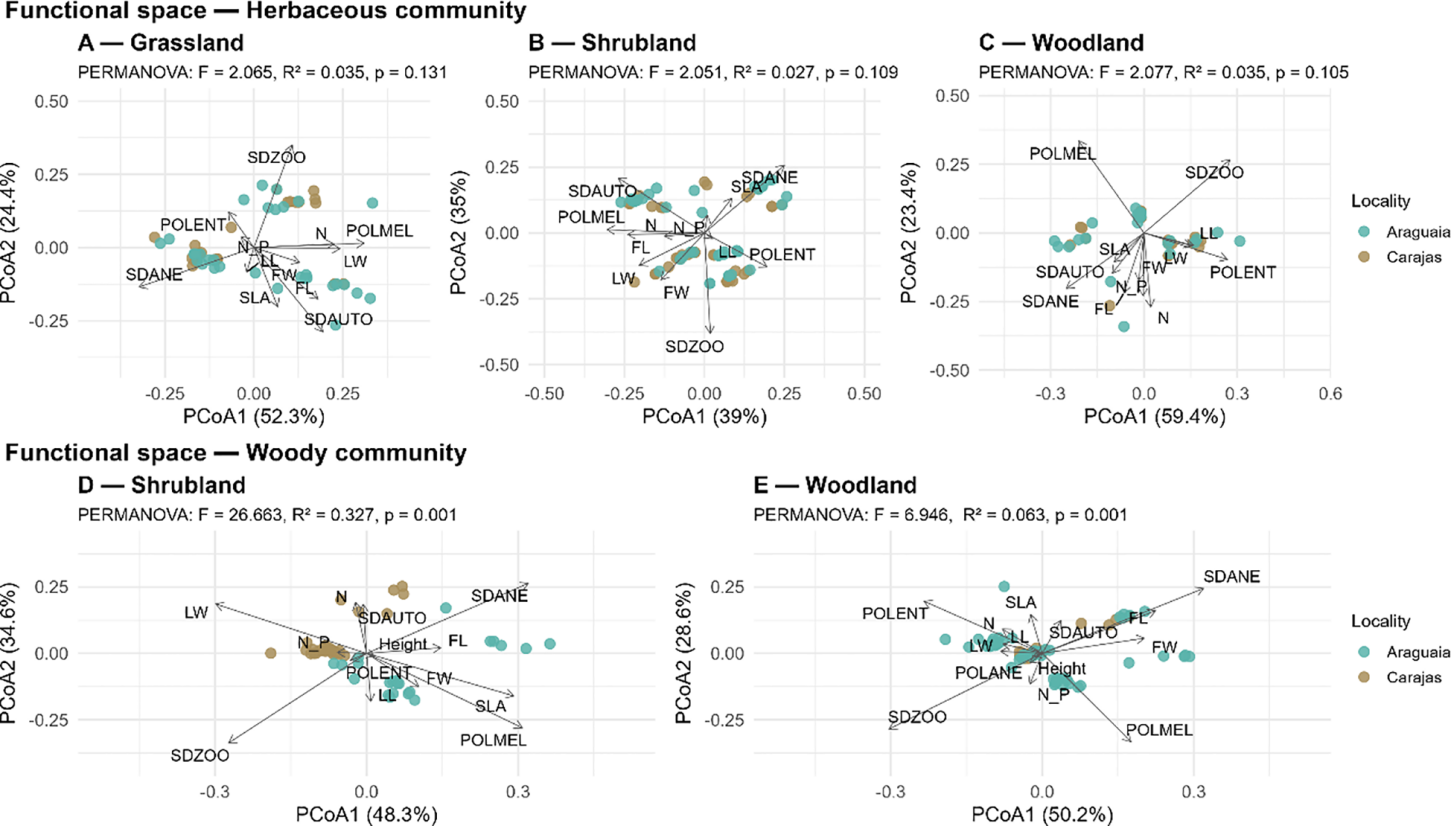
Principal coordinate analysis (PCoA) based on functional traits of the herbaceous: **(A)** Grassland, **(B)** Shrubland, and **(C)** Woodland; and woody community **(D)** Shrubland and **(E)** Woodland. LL (leaf length), LW (leaf width), FL (flower length), FW (flower width), N (leaf nitrogen), N:P (nitrogen-phosphorus ratio), SLA (specific leaf area), Height (plant height), SDAUTO (autochorous dispersal), SDZOO (zoochorous dispersal), SDANE (anemochorous dispersal), POLENT (entomophilous pollination), POLMEL (melittophilous pollination), and POLANE (anemophilous pollination). Carajás in brown; Lower Araguaia River basin in teal.

Despite the floristic dissimilarity between regions, principal coordinate analysis (PCoA) revealed overlapping functional trait spaces between regions. For the herbaceous component, no significant differences were observed between Carajás and Araguaia in the GS, SB, and WD formations (p > 0.05; **Figures 4A–C**), indicating functional convergence across regions. The first PCoA axis reflected a resource acquisition and use gradient, predominantly associated with leaf and nutritional traits (LL, LW, N, N:P), whereas the second axis represented reproductive strategies related to dispersal and pollination syndromes. In contrast, the woody community showed significant functional differences between regions in the SB and WD formations (p = 0.001; **Figures 4D, E**). In this stratum, the first axis was structured primarily by height and reproductive traits, whereas the second axis was influenced by leaf and nutrient-related traits.

## 4 Discussion

Our study revealed floristic and functional patterns in ferruginous outcrops from the Lower Araguaia basin and Carajás. Although both regions host similar vegetation formations, they occur under distinct climatic, geological, and geochemical contexts. In the Araguaia region the climate is a warmer, with lower annual rainfall and a more prolonged dry season. These conditions increase vegetation flammability, making natural fires an important factor influencing plant community organization (Sanjuan et al., 2025). Geologically, Araguaia ferruginous outcrops exhibit a more heterogeneous mineralogy, influenced by mafic–ultramafic lithotypes enriched in Ni and Cr, as well as a greater contribution of silica (Barros and De Sousa Gorayeb, 2019; Guimarães et al. In Prep.). In contrast, Carajás, ferruginous outcrops are characterized by high concentrations of Fe and Al oxides, which intensify phosphorus limitations. Despite these geological and geochemical differences, both systems develop shallow, nutrient-poor, and acidic soils and share a comparable environmental gradient that ranges from open and shallow grasslands (GS) to more structurally complex shrublands (SB) and woodlands (WD). This gradient could be associated with abiotic factors such as soil depth, nutrient availability, and solar exposure, which are commonly reported as environmental filters in ferruginous ecosystems (Castro et al. In Prep), potentially sorting species along the gradient and helping to explain differences in plant functional strategies across formations.

The floristic overlap observed between ferruginous outcrops from both regions indicates a shared species pool, although floristic differences further highlight the importance of regional peculiarities (Zappi et al., 2019). The latter may be explained by the geographic position of the Araguaia within the Amazon–Cerrado ecotone. This transitional setting between two major biomes would promote the coexistence of species with different biogeographic origins, thereby increasing compositional variability and reinforcing regional differentiation in plant communities. However, it is important to note that a complete floristic checklist for the ferruginous outcrops of the Lower Araguaia River basin region is not yet available. This limitation prevents a comprehensive assessment of the degree of floristic overlap between regions, restricting the comparison to the most dominant species recorded in this study relative to the complete species list from the Carajás *cangas* (Mota et al., 2018).

The richness patterns showed formation-specific contrasts. In GS, richness was lower in the Araguaia region than in Carajás, which can be attributed to the greater configurational heterogeneity and microtopographic ruggedness reported for Carajás (Gastauer et al., 2021), increasing the number of ecological niches involved in GS formation. In SB, however, richness did not differ between regions. This pattern may result from the higher vertical complexity and deeper soils typical of SB formations, combined with the contribution of ecotonal species pools in the Araguaia, which together can buffer regional differences in landscape heterogeneity. These results suggest that while microhabitat heterogeneity enhances richness in open herbaceous formations in Carajás, SB formations, which are structurally more complex, have species richness that is more strongly influenced by vegetation structure and by the regional availability of species than by local topographic variation. In the Araguaia region, the presence of continuous Cerrado areas may expand the regional pool of shrub species, contributing to species richness. In the WD of Carajás, greater species richness was observed than in the Araguaia region. In this region, plants appear to adopt efficient strategies of nutrient conservation and internal nutrient cycling, possibly associated with a greater reliance on mycorrhizal associations (Castro et al. In Prep), contributing to sustaining high levels of species richness. Additionally, this pattern may also be related to the Amazonian context, which likely provides a broader regional tree species pool (Ron et al., 2018).

In the herbaceous community from GS and SB in both regions, leaf trait patterns further indicate strong environmental filtering and the coexistence of stress-tolerant species (Poorter, 2009; Silveira et al., 2016). In Carajás, lower SLA values indicate slightly more conservative resource-use strategies. The lower foliar N and observed low N:P ratios in GS and SB across both regions are consistent with nutrient limitation and the dominance of stress-tolerant strategies (Perez-Harguindeguy et al., 2013; Araújo et al., 2023), both typical of ferruginous environments. These functional patterns may be associated with the edaphic context. Although some soil nutrients vary between regions, the results indicate that both have low-fertility soils, especially with respect to P, showing deficiency of this nutrient across all formations in both regions. The GS formations of Carajás tend to have more clay-rich soils than those of Araguaia. Clayey soils developed over Fe and Al-rich substrates exhibit high adsorption capacity, promoting strong retention and immobilization of P and other nutrients (Yi et al., 2023; Veloso et al., 2023). Such limitations restrict nutrient availability to plants and may increase the ecological importance of symbiotic associations that enhance nutrient acquisition (Monteiro et al., 2022; Martin and van der Heijden, 2024). In contrast, in the WD of the Araguaia, higher foliar nitrogen contents were observed, despite lower soil N and P levels. This pattern suggests that species adapted to nutrient-poor environments tend to invest proportionally more foliar N to maintain metabolic function, whereas P may be more strongly remobilized from senescent tissues (Mayor et al., 2014; Estiarte et al., 2023).

In the woody community (SB, WD), functional patterns indicated greater between-region divergence, which was consistent with niche differentiation in more structurally complex environments. For example, Araguaia SB presented high SLA coupled with larger fruit size, suggesting a strategy that combines rapid resource acquisition with reproductive investment under limiting conditions (Metz et al., 2023; Dombroskie et al., 2016), whereas SB woody community in Carajás presented greater height, greater leaf N, and elevated N:P, indicative of less severe nutrient constraints. In WD, Araguaia ferruginous outcrops retained more open-structure functional traits, including greater fruit width and higher N:P, which is consistent with greater P limitation (Sandoval-Granillo and Meave, 2023), whereas Carajás WD developed broader leaves to maximize light capture under closed canopies.

The greater proportion of abiotic dispersal and pollination observed in the Araguaia outcrops may reflect the ecological context of the Amazon-Cerrado ecotone, which is characterized by open savanna mosaics and pronounced seasonality. In such environments, wind dispersal is common during the late rainy and dry seasons (Kuhlmann and Ribeiro, 2016; Escobar et al., 2018), indicating that open and seasonal habitats favor this type of reproductive strategy. In contrast, Carajás outcrops occur as island-like habitats embedded in a dense forest matrix (Giannini et al., 2021). The combination of high topographic roughness, mountainous terrain, and deep valleys, may increase the dependence on biotically mediated dispersal, particularly at intermediate spatial scales between isolated outcrop patches. The maintenance of biotic interaction networks, even under strong spatial isolation (Pinto et al., 2020), underscores the role of animal vectors in sustaining ecological connectivity among Carajás outcrops.

Taken together, these trends indicate that although ferruginous ecosystems in the Araguaia and Carajás are species rich and share part of their floristic pool, they also exhibit compositional differences influenced by the mosaic nature and spatial disjunction of ferruginous substrates. Despite this floristic turnover, communities in open herbaceous formations (GS and SB) show marked structural and functional similarity across regions, suggesting similar ecological strategies. Moreover, floristic similarity and moderate functional similarity reveal substantial trait overlap between regions (Ricotta and Pavoine, 2022; Rigou et al., 2022; Ricotta and Podani, 2017), indicating that the GS and SB plant communities from the Araguaia and Carajás occupy similar ecological niches. In contrast, the pronounced functional divergence in the woody communities reflects the influence of distinct ecological processes in structurally more complex environments.

## 5 Conclusion

In conclusion, our study highlights the floristic uniqueness and similarities of ferruginous outcrop ecosystems in the Lower Araguaia River basin and Carajás, emphasizing their functional convergence. Despite occurring in distinct climatic and geological contexts, both regions possess nutrient-poor soils, particularly characterized by strong P limitation. Even so, the ferruginous outcrops from both regions share an environmental gradient that shapes species functional traits and ecological strategies between open vegetation formations. Our results indicate that open formations of the ferruginous outcrops of the Araguaia exhibit functional equivalence to those in Carajás, whereas notable differences in woody communities underscore the need for tailored conservation approaches. Given the growing threats from agricultural expansion in the Araguaia region, it is crucial to implement strategies that both safeguard the unique floristic compositions of each region and leverage the functional equivalence of open formations, thereby promoting the long-term persistence of ferruginous outcrop ecosystems in the Eastern Amazon.

## Data Availability

The original contributions presented in the study are included in the article/**Supplementary Material**. Further inquiries can be directed to the corresponding author.
