# Supplementary material for: Environmental filters drive functional similarity in disjunct ferruginous outcrops of Eastern Amazonia
Source: Front Plant Sci. 2026 Jan 28;16:1695218. doi: 10.3389/fpls.2025.1695218 (PMC12891204; doi:10.3389/fpls.2025.1695218)
Supplement: Supplementary file 1 [file DataSheet1.docx]

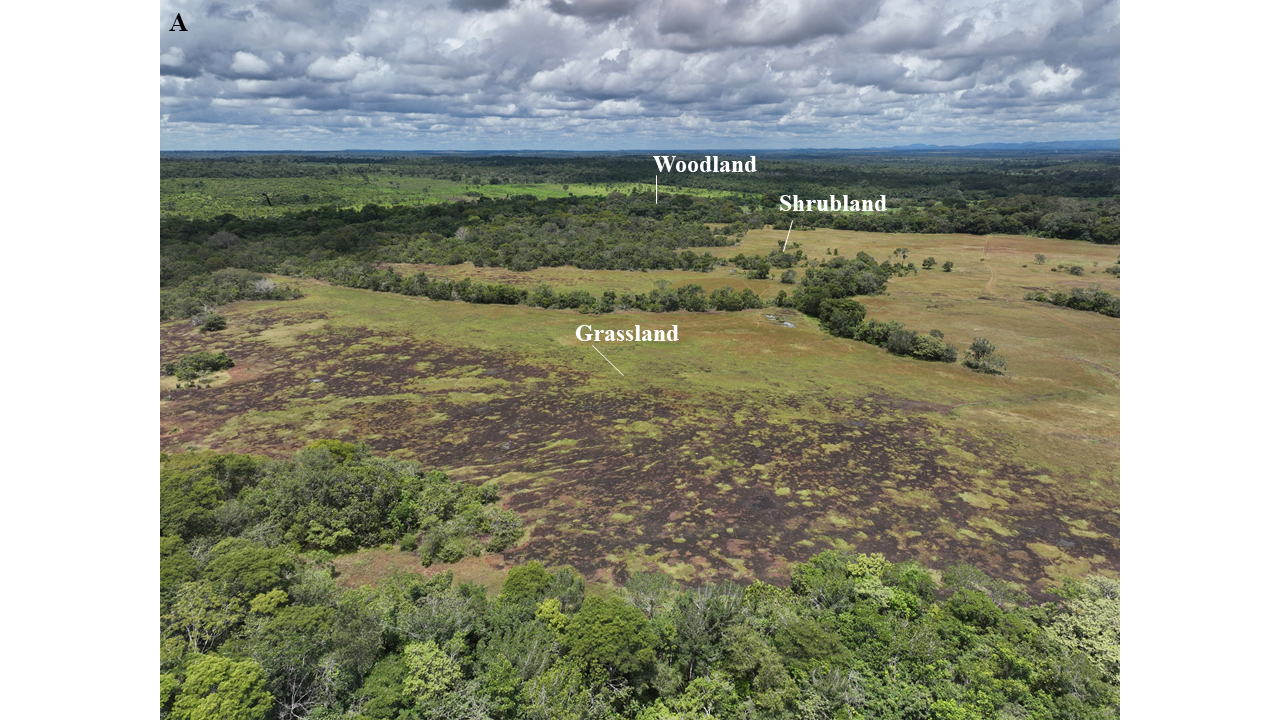

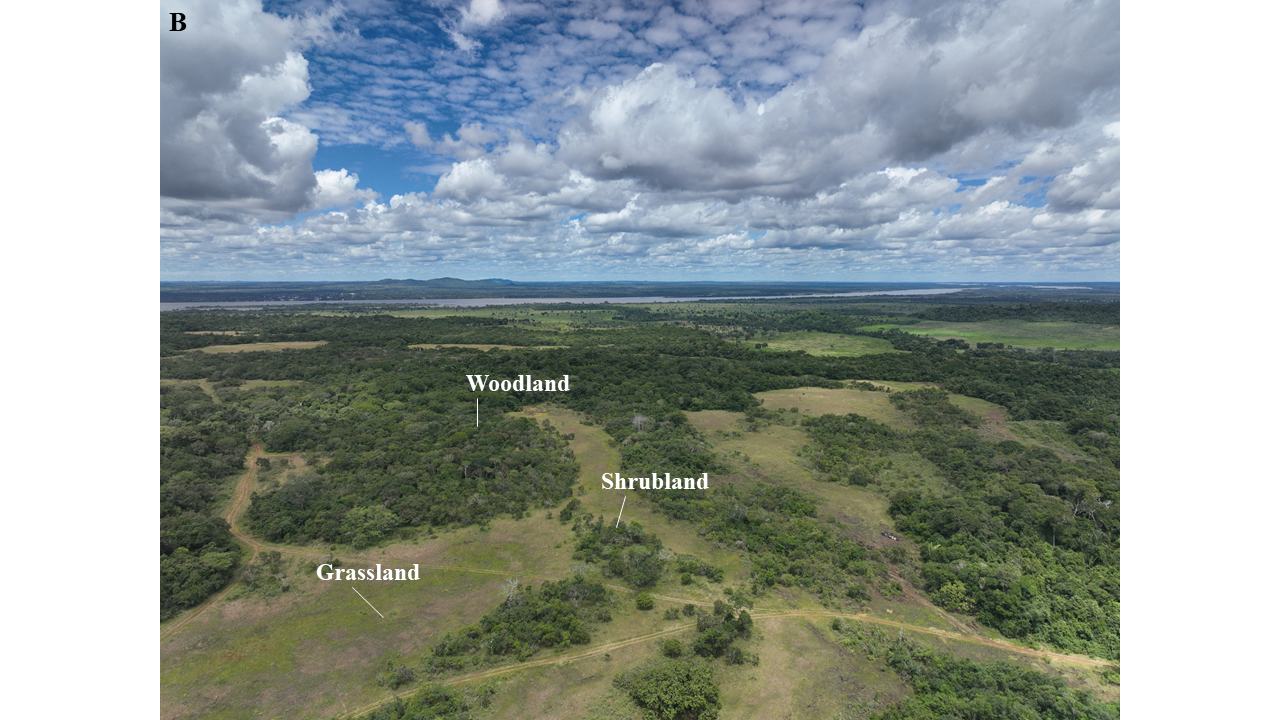
**SUPPLEMENTARY MATERIAL**

**Fig S1.** Aerial photographs of the vegetation formations sampled in Carajás (A) and the Floresta do Araguaia municipality from the Lower Araguaia River basin (B).

**
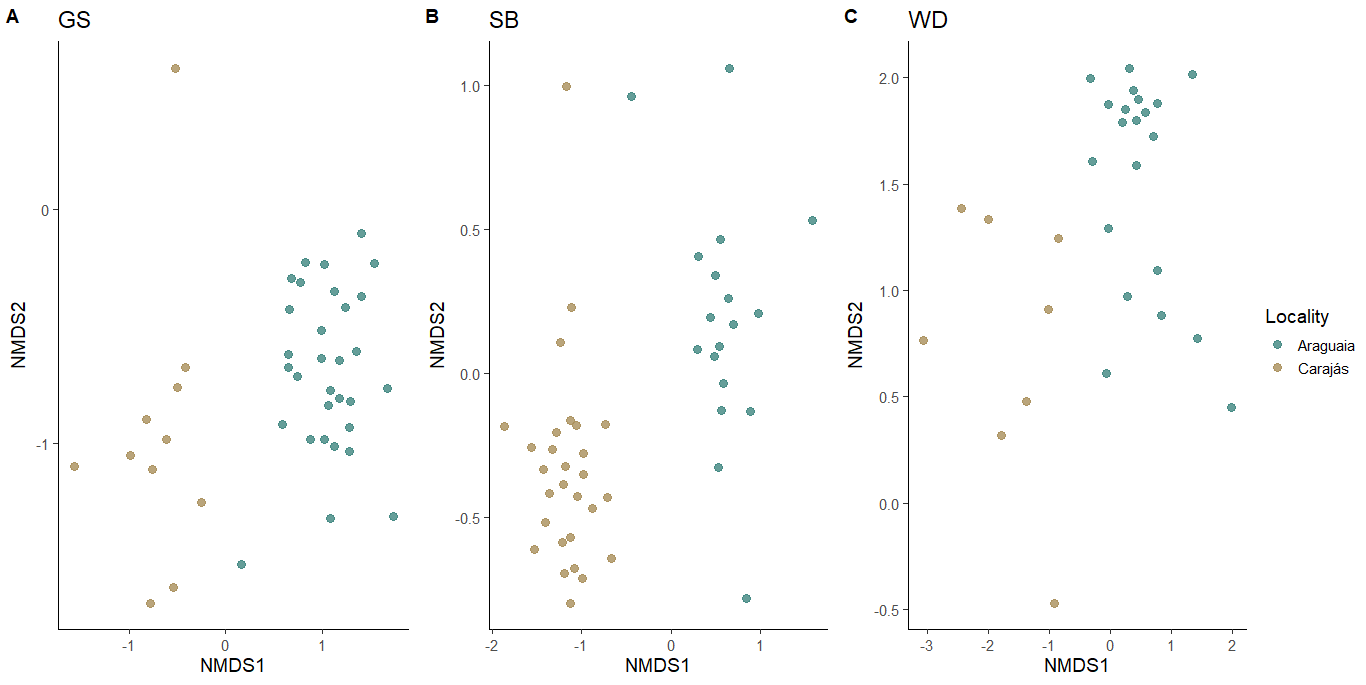
**

**Fig. S2.** Nonmetric multidimensional scaling (NMDS) ordination based on species composition across vegetation formations: GS (grassland), SB (shrubland), and WD (woodland).


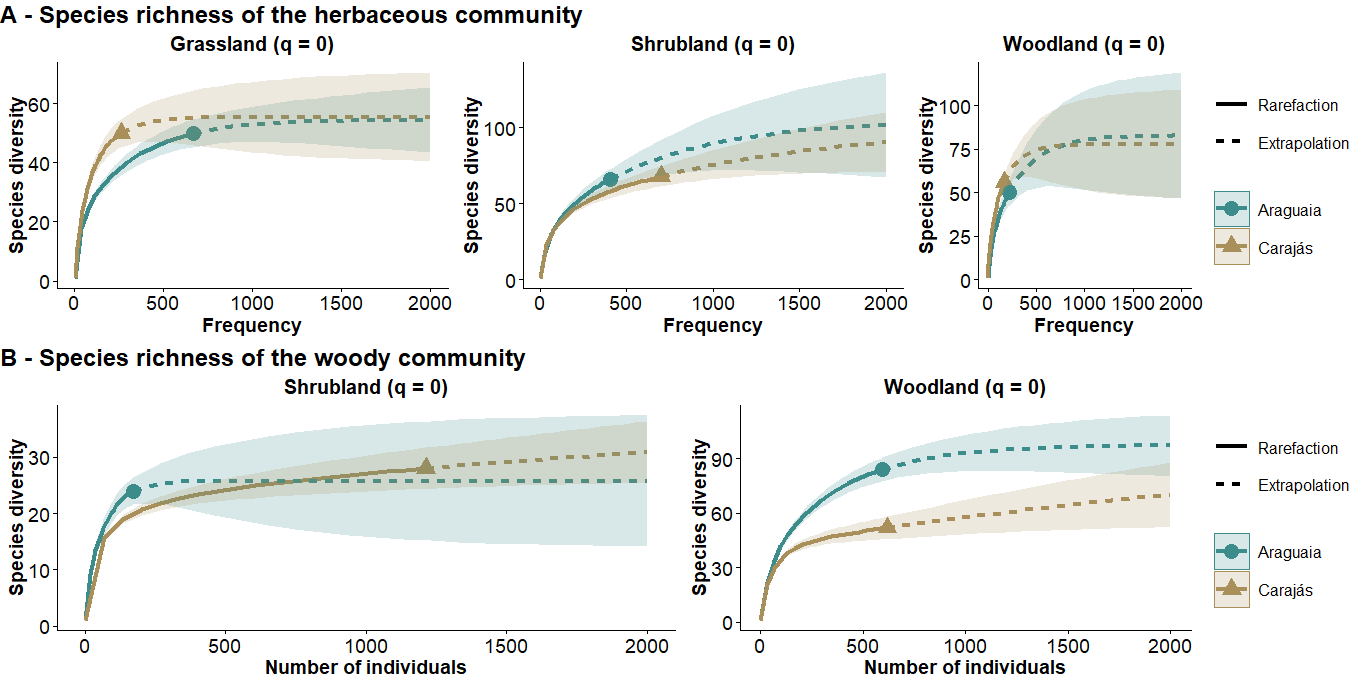


**Fig. S3.** Rarefaction and extrapolation curves of species richness for the different formations of ferruginous outcrops from Carajás (brown) and from the Lower Araguaia River basin (teal). A – Herbaceous community; B – Woody community


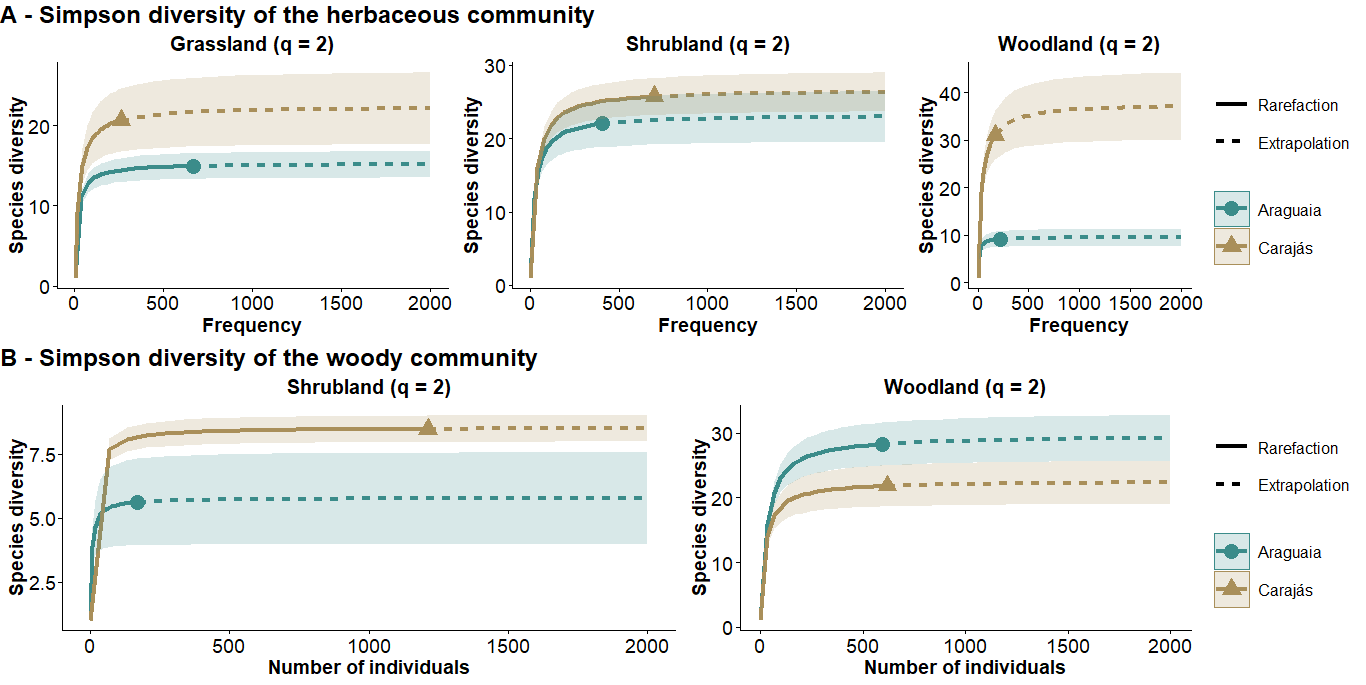


**Fig. S4.** Rarefaction and extrapolation curves of Simpson diversity for the different formations of ferruginous outcrops from Carajás (brown) and from the Lower Araguaia River basin (teal). A – Herbaceous community; B – Woody community.


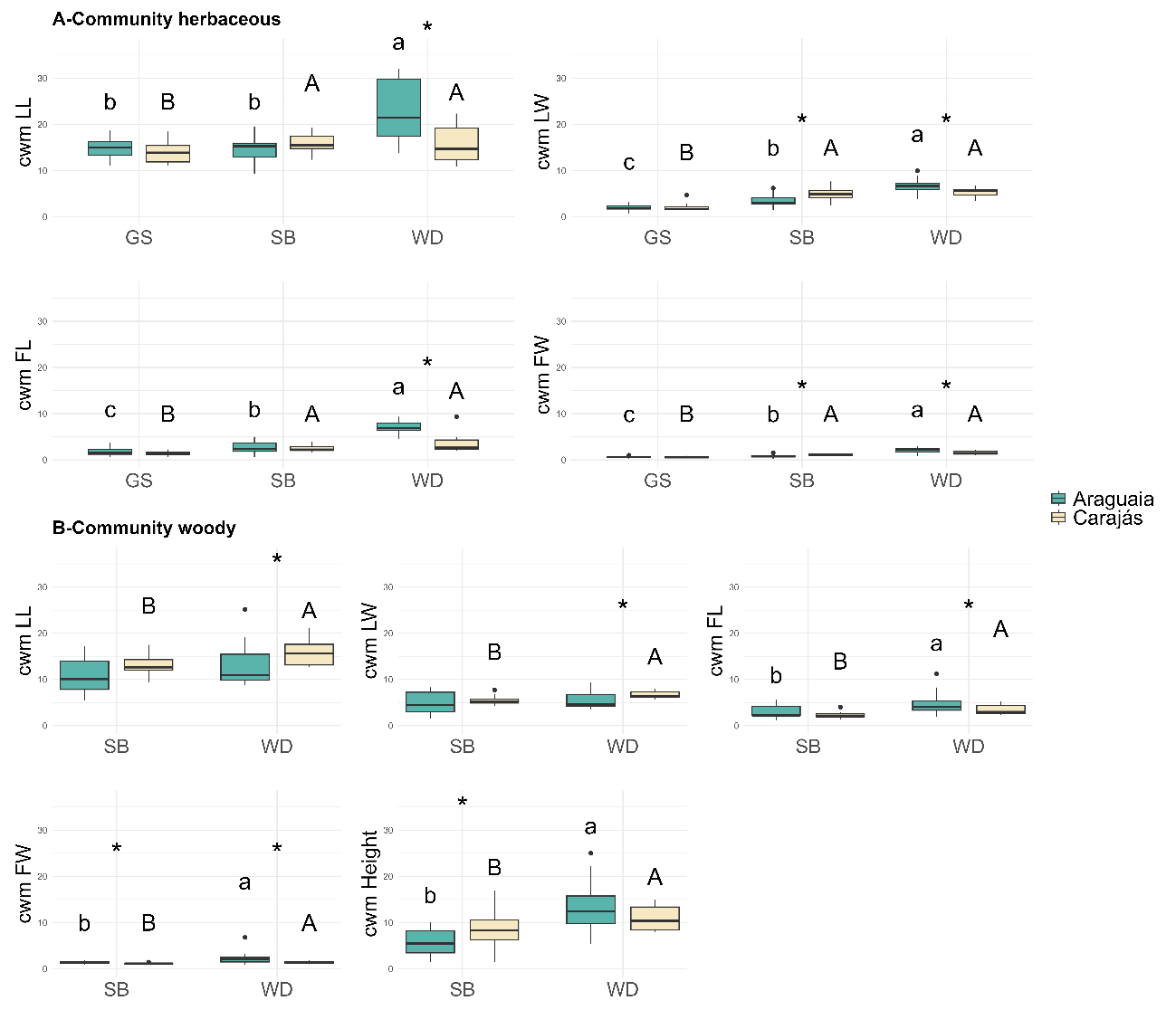


**Fig. S5.** Community-weighted mean (CWM) values of species traits of different vegetation formations from ferruginous outcrops of Carajás and the Lower Araguaia River basin: **A** - Herbaceous and **B** - Woody communities: LL – leaf length; LW – leaf width; FL – fruit length; FW - fruit width; Height). Different lowercase letters indicate significant differences among the Carajás formations; uppercase letters indicate significant differences among the Aragauia basin formations; (*) indicates significant differences between localities (p < 0.05). GS is Grassland, SB is Shrubland and WD is Woodland.


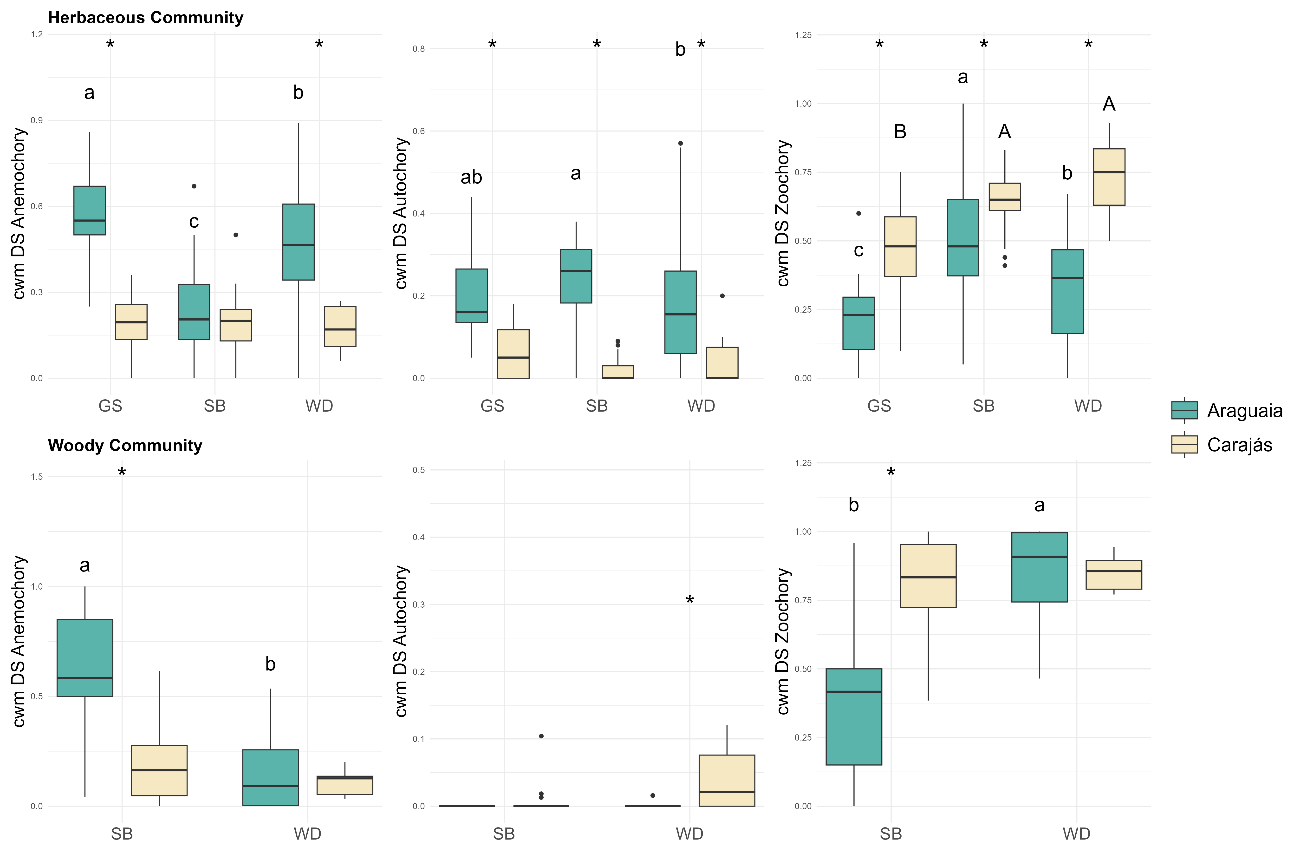


**Fig. S6.** Community-weighted mean (CWM) values of species traits and dispersal syndromes of different vegetation formations from ferruginous outcrops in Carajás and the Lower Araguaia River basin: A – Herbaceous strata and B – Woody communities. Different lowercase letters indicate significant differences among the Carajás formations; uppercase letters indicate significant differences among the Araguaia basin formations; (*) indicates significant differences between localities (p < 0.05). GS = Grassland, SB = Shrubland, WD = Woodland.


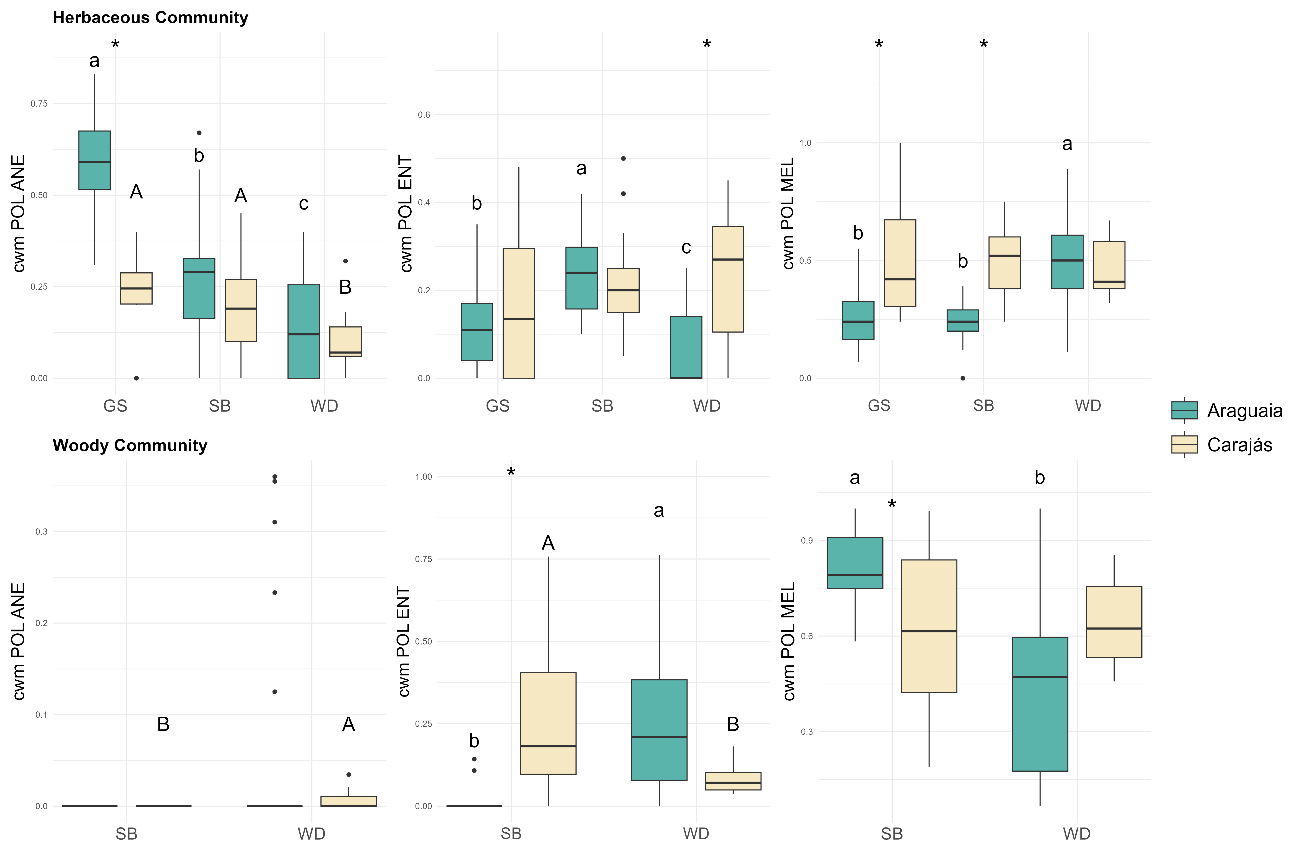


**Fig. S7.** Community-weighted mean (CWM) values of pollination syndromes in different vegetation formations from ferruginous outcrops in Carajás and the Lower Araguaia River basin: A – Herbaceous and B – Woody communities. Different lowercase letters indicate significant differences among the Carajás formations; uppercase letters indicate significant differences among the Araguaia basin formations; (*) indicates significant differences between localities (p < 0.05). GS = Grassland, SB = Shrubland, WD = Woodland.

**Table S1**. Summary of the main community‐weighted functional traits (means ± SDss) with the highest ecological relevance, describing the resource‐use strategies of plant communities from ferruginous outcrops in Carajás and the Lower Araguaia River basin (Eastern Amazonia, Brazil).

| **Locality** | **Communities** | **Physiognomy** | **SLA (cm²/g)** | **N:P ratio** | **Leaf N (g/kg)** |
| --- | --- | --- | --- | --- | --- |
|  |  |  | *Mean ± SD* | *Mean ± SD* | *Mean ± SD* |
| **Araguaia** | Herbaceous | GS | 321.34 ± 100.31 | 18.76 ± 4.51 | 15.53 ± 1.91 |
|  |  | SB | 374.88 ± 86.11 | 19.72 ± 6.64 | 17.96 ± 3.87 |
|  |  | WD | 700.50 ± 174.86 | 43.38 ± 16.20 | 33.15 ± 11.50 |
| **Carajás** | Herbaceous | GS | 164.71 ± 45.05 | 22.53 ± 2.25 | 15.36 ± 1.25 |
|  |  | SB | 184.11 ± 30.69 | 18.10 ± 4.18 | 17.99 ± 2.98 |
|  |  | WD | 521.51 ± 212.70 | 21.01 ± 1.71 | 17.79 ± 1.27 |
| **Araguaia** | Woody | SB | 501.54 ± 34.84 | 26.48 ± 2.57 | 13.34 ± 1.66 |
|  |  | WD | 453.92 ± 106.46 | 25.61 ± 4.87 | 16.80 ± 1.79 |
| **Carajás** | Woody | SB | 118.27 ± 16.02 | 25.09 ± 4.20 | 19.73 ± 3.91 |
|  |  | WD | 126.75 ± 8.22 | 21.64 ± 2.08 | 16.63 ± 1.26 |

**Table S2.** Results of Wilcoxon tests comparing the CWM values of leaf traits between Carajás and Araguaia across physiognomies and communities.

| **Communities** | **Traits** | **Physiognomies** | **p value** | **adjusted p value** | **Sig.** |
| --- | --- | --- | --- | --- | --- |
| **Herbaceous** | SLA | GS | 3.02×10⁻⁵ | 4.54×10⁻⁵ | *** |
| **Herbaceous** | SLA | SB | 4.38×10⁻¹³ | 1.31×10⁻¹² | *** |
| **Herbaceous** | SLA | WD | 3.08×10⁻² | 3.08×10⁻² | * |
| **Herbaceous** | N:P | GS | 1.51×10⁻¹ | 2.27×10⁻¹ | ns |
| **Herbaceous** | N:P | SB | 7.53×10⁻¹ | 7.53×10⁻¹ | ns |
| **Herbaceous** | N:P | WD | 2.25×10⁻⁶ | 6.76×10⁻⁶ | *** |
| **Herbaceous** | N | GS | 4.21×10⁻¹ | 6.32×10⁻¹ | ns |
| **Herbaceous** | N | SB | 8.44×10⁻¹ | 8.44×10⁻¹ | ns |
| **Herbaceous** | N | WD | 4.28×10⁻⁵ | 1.28×10⁻⁴ | *** |
| **Woody** | SLA | SB | 1.23×10⁻⁸ | 2.45×10⁻⁸ | *** |
| **Woody** | SLA | WD | 3.42×10⁻⁷ | 3.42×10⁻⁷ | *** |
| **Woody** | N:P | SB | 2.34×10⁻¹ | 2.34×10⁻¹ | ns |
| **Woody** | N:P | WD | 1.56×10⁻² | 3.11×10⁻² | * |
| **Woody** | N | SB | 4.40×10⁻⁶ | 8.81×10⁻⁶ | *** |
| **Woody** | N | WD | 8.01×10⁻¹ | 8.01×10⁻¹ | ns |

**Table S3.** Species from ferruginous outcrops of the Lower Araguaia River basin (n = 174) and their occurrence in Carajás (Mota et al., 2018).

| **Families** | **Species** | **Occurrence in**  **Carajás** |
| --- | --- | --- |
| Anacardiaceae | *Anacardium occidentale* L. | Yes |
| Anacardiaceae | *Thyrsodium spruceanum* Benth. | Yes |
| Anacardiaceae | *Spondias mombin* L. | No |
| Annonaceae | *Annona coriacea* Mart*.* | No |
| Annonaceae | *Annona exsuca* DC. | No |
| Annonaceae | *Annona neoinsings* H.Rainer | No |
| Annonaceae | *Duguetia riparia Huber* | No |
| Annonaceae | *Guateria punctata* (Aubl.) R.A.Howard | Yes |
| Annonaceae | *Guateria sp* Ruiz & Pav. | No |
| Annonaceae | *Xylopia aromatica* (Lam.) Mart | Yes |
| Annonaceae | *Xylopia serecia* A.St.-Hil | No |
| Apocynaceae | *Apisdosperma desmanthum* Benth. Ex Mull. Arg | No |
| Apocynaceae | *Apisdosperma macrocarpo* Mart. & Zucc. | No |
| Apocynaceae | *Aspidosperma subincanum* Mart. ex A.DC. | Yes |
| Apocynaceae | *Himatanthus articulatus* (Vahl) Woodson | No |
| Apocynaceae | *Himatanthus obovatus* (Muell.Arg.) Woodson | No |
| Arecaceae | *Attalea maripa* (Aubl.) Mart. | Yes |
| Arecaceae | *Oenocarpus distichus* Mart. | Yes |
| Arecaceae | *Syagrus cocoides* Mart. | Yes |
| Asteraceae | *Bidens pilosa* L. | Yes |
| Asteraceae | *Cavalcantia percymosa* R.M.King & H.Rob. | Yes |
| Asteraceae | *Lepidaploa remotiflora* (Rich.) H.Rob. | Yes |
| Asteraceae | *Riencourtia pedunculosa* (Rich.) Pruski | Yes |
| Bignoneaceae | *Handroanthus sp* Mart. | No |
| Bignoneaceae | *Handroanthus serratifolius* (Valh) S.Grose | No |
| Bignoniaceae | *Pleonotoma jasminifolia* (S.Moore) A.H.Gentry | No |
| Bignoneaceae | *Pleonotoma melioides* (S.Moore) A.H.Gentry | Yes |
| Bignoniaceae | *Pleonotoma orientalis* Sandwith | Yes |
| Bixaceae | *Cochlospermum orinocense* (Kunth) Steud. | Yes |
| Bromeliaceae | *Ananas ananassoides* (Baker) L.B.Sm | Yes |
| Burseraceae | *Protium decandrum* (Aubl.) Marchand | No |
| Burseraceae | *Protium heptaphyllum* (Aubl.) Marchand | No |
| Burseraceae | *Protium pilosissimum* Engl | Yes |
| Burseraceae | *Protium subserratum*  (Engl.) Engl. | No |
| Chrysobalanaceae | *Hirtella racemosa* Lam | Yes |
| Chrysobalanaceae | *Hirtella sp* L. | No |
| Chrysobalanaceae | *Licania alba* (Bernoulli) Cuatrec. | No |
| Chrysobalanaceae | *Licania canescens* Benoist | No |
| Combretaceae | *Combretum laxum* Jacq | Yes |
| Connaraceae | *Connarus perrotteii* (DC.) Planch. | Yes |
| Connaraceae | *Rourea induta* Planch. | Yes |
| Convolvulaceae | *Evolvulus filipes* Mart. | No |
| Cordiaceae | *Cordia sp* L. | No |
| Costaceae | *Chamaecostus lanceolatus* (Petersen) C.D.Specht & D.W.Stev. | No |
| Cyperaceae | *Bulbostylis conifera* C.S.Nunes & A.Gil | Yes |
| Cyperaceae | *Cyperus aggregatus* (Willd.) Endl. | Yes |
| Cyperaceae | *Rhynchospora acanthoma* A.C.Araújo &  Longhi-Wagner | Yes |
| Cyperaceae | *Rhynchospora aff secoi* C.S. Nunes, P.J.S. Silva Filho  & A. Gil | No |
| Cyperaceae | *Rhynchospora* *barbata* (Vahl) Kunth | Yes |
| Cyperaceae | *Rhynchospora* *filiformis* Vahl | Yes |
| Cyperaceae | *Rhynchospora unguinux* C.S.Nunes & A.Gil | No |
| Erythropalaceae | *Heisteria ovata* | No |
| Erythroxylaceae | *Erythroxylum carajasense* (Plowman) Costa-Lima | Yes |
| Erythroxylaceae | *Erythroxylum squamatum* Sw | Yes |
| Erythroxylaceae | *Erythroxylum subracemosum* Turcz. | Yes |
| Erythroxylaceae | *Erythroxylum sp* L. | Yes |
| Euphorbiaceae | *Alchornea discolor* Poepp | Yes |
| Euphorbiaceae | *Mabea fistulifera* Mart. | No |
| Euphorbiaceae | *Mabea sp* Mart. | No |
| Euphorbiaceae | *Maprounea guianensis* Aubl. | No |
| Euphorbiaceae | *Microstachys glandulosa* (Mart.) F.Dietr. | No |
| Euphorbiaceae | *Sapium glandulosum* (L.) Morong | No |
| Fabaceae | *Aeschynomene americana* Hard glandulosa (Poir.)  Rudd | Yes |
| Fabaceae | *Anadenanthera columbrina (L.) Speg.* | No |
| Fabaceae | *Anadenanthera peregrina* (L.) Speg. | Yes |
| Fabaceae | *Andira surinamensis* (Bondt) Splitg. ex Amshoff | No |
| Fabaceae | *Bauhinia brevips* | No |
| Fabaceae | *Bauhinia longicuspis* Benth. | Yes |
| Fabaceae | *Chamaecrista flexuosa* (L.) Greene | No |
| Fabaceae | *Chloroleucon tortum* (Mart.) Pittier | No |
| Fabaceae | *Copaifera langsdorfii* Desf. | No |
| Fabaceae | *Crotalaria maypurensis* Kunth | Yes |
| Fabaceae | *Crotalaria sp* L. | No |
| Fabaceae | *Dimorphandra mollis* Benth | No |
| Fabaceae | *Galactia jussiaeana* Kunth | Yes |
| Fabaceae | *Hymenaea courbaril* L. | No |
| Fabaceae | *Inga sp* L. | No |
| Fabaceae | *Machaerium sp L.* | No |
| Fabaceae | *Mimosa hirsutissima* Mart. | No |
| Fabaceae | *Mimosa skinneri var. desmodioides* Mart. | No |
| Fabaceae | *Senegalia polyphylla (DC.)* Britton & Rose | No |
| Fabaceae | *Senna macranthera* (DC. ex Collad.) H.S.Irwin &  Barneby | Yes |
| Fabaceae | *Stryphnodendron adstringens* (Mart.) Coville | No |
| Fabaceae | *Stryphnodendron cf hirwin* L. | No |
| Fabaceae | *guianense* (Aubl.) Benth. | No |
| Fabaceae | *Stryphnodendron pulcherrimum* (Willd.) Hochr. | Yes |
| Fabaceae | *Tachigali vulgaris* L.G.Silva & H.C.Lim | Yes |
| Fabaceae | *Tachigali paniculata* Aubl | No |
| Gentianaceae | *Schultesia benthamiana* Klotzsch ex Griseb | Yes |
| Gentinaceae | *Schultesia guianensis* (Aubl.) Malme var. guianensis | No |
| Hypericaceae | *Vismia cayennensis* (Jacq.) Pers | Yes |
| Humiriaceae | *Endopleura uchi* (Huber) Cuatrec. | No |
| Iridaceae | *Cipura xanthomelas* Maxim ex Klatt | Yes |
| Lythraceae | *Cuphea annulata* Koehne | Yes |
| Lythraceae | *Cuphea sp* P.Browne | No |
| Lythraceae | *Cuphea tenuissima* Koehne | No |
| Malpighiaceae | *Banisteriopsis malifolia* (Nees ex Mart.) B.Gates | Yes |
| Malpighiaceae | *Banisteriopsis* *stellaris* (Griseb.) B.Gates | Yes |
| Malpighiaceae | *Banisteriopsis sp* C.B.Rob. ex Small | No |
| Malpighiaceae | *Byrsonima chrysophylla* Kunth | Yes |
| Malpighiaceae | *Byrsonima sp* Rich. ex Kunth | No |
| Malvaceae | *Apeiba tiborbou* Aubl. | No |
| Malvaceae | *Ceiba pentandra* (L.) Gaertn. | No |
| Malvaceae | *Pachira sp* L. | No |
| Marantaceae | *Ischnosiphon sp* Korn | No |
| Melastomataceae | *Miconia albicans* (Sw.) Steud. | Yes |
| Melastomataceae | *Miconia phyllitropioides* (DC.) Naudin | No |
| Melastomataceae | *Miconia sp* Ruiz & Pav. | No |
| Melastomataceae | *Mouriri ellipitica* Mart. | No |
| Melastomataceae | *Mouriri pusa* Gardner | No |
| Melastomataceae | *Mouriri sp* Aubl. | No |
| Menispermaceae | *Abuta grandifolia* (Mart.) Sandwith | Yes |
| Moraceae | *Maclura tinctoria* (L.) D.Don ex Steud. | No |
| Myristicaceae | *Virola sebifera* Aubl. | No |
| Myrtaceae | *Eugenia chrysophyllum* Poir*.* | No |
| Myrtaceae | *Eugenia involucrata* DC. | No |
| Myrtaceae | *Eugenia punicifolia* (Kunth) DC | Yes |
| Myrtaceae | *Eugenia sp* L. | No |
| Myrtaceae | *Myrcia splendens* (Sw.) DC | Yes |
| Myrtaceae | *Myrcia sp* DC | Yes |
| Nyctaginaceae | *Neea ovalifolia* Spruce ex J.A.Schmidt | No |
| Ochnaceae | *Ouratea castaneifolia* (DC.) Engl. | Yes |
| Ochnaceae | *Sauvagesia tenella* Lam | Yes |
| Olacaceae | *Heisteria ovata* Benth. | Yes |
| Olacaceae | *Ximenia americana* L | Yes |
| Orchidaceae | *Habenaria sp* Willd. | No |
| Orobanchaceae | *Buchnera carajasensis* Scatigna & N.Mota | Yes |
| Phyllanthaceae | *Margaritaria nobilis* L.f | No |
| Piperaceae | *Peperomia albopilosa* D.Monteiro | Yes |
| Poaceae | *Andropogon bicornis* L | Yes |
| Poaceae | *Aristida sp* Lam | No |
| Poaceae | *Axonopus aureus* P.Beauv | Yes |
| Poaceae | *Axonopus* *capillaris* (Lam.) Chase | Yes |
| Poaceae | *Axonopus* *rupestris* Davidse | Yes |
| Poaceae | *Axonopus sp* P. Beauv. | No |
| Poaceae | *Mesosetum annuum* Swallen | Yes |
| Poaceae | *Mesosetum cayennense* Steud. | Yes |
| Poaceae | *Mesosetum* *filifolium* F.T.Hubb. | Yes |
| Poaceae | *Mesosetum sp* Steud. | No |
| Poaceae | *Pariana caxiuanensis* Aubl. | No |
| Poaceae | *Paspalum carajasense* S.Denham | Yes |
| Poaceae | *Paspalum expansum* Döll | Yes |
| Poaceae | *Paspalum lanciflorum* Trin. | Yes |
| Poaceae | *Paspalum melanospermum* Desv. ex Poir | Yes |
| Poaceae | *Paspalum multicaule* Poir | Yes |
| Poaceae | *Paspalum aff spissum* Swallen | No |
| Poaceae | *Paspalum sp* L | No |
| Poaceae | *Trachypogon spicatus* (L.f.) Kuntze | Yes |
| Polygalaceae | *Bredemeyera parviflora* Spruce ex A.W.Benn. | No |
| Polygalaceae | *Senega adenophora* Spach | No |
| Polygalaceae | *Senega chapadensis* Spach | No |
| Portulacaceae | *Portulaca sedifolia* N.E.Br | Yes |
| Rubiaceae | *Borreria heteranthera* E.L.Cabral & Sobrado | Yes |
| Rubiaceae | *Borreria semiamplexicaulis* E.L.Cabral | Yes |
| Rubiaceae | *Borreria sp* G. Mey | No |
| Rubiaceae | *Chomelia ribesioides* Benth. ex A.Gray | Yes |
| Rubiaceae | *Mitracarpus carajasensis* E.L.Cabral, Sobrado & E.B.Souza | Yes |
| Rubiaceae | *Mitracarpus sp Zucc. ex Schult. & Schult.f.* | No |
| Rubiaceae | *Tocoyena formosa* (Cham. & Schltdl.) K.Schum. | Yes |
| Rutaceae | *Zanthoxylum rhoifolium* Lam. | No |
| Salicaceae | *Casearia arborea* (Rich.) Urb | Yes |
| Salicaceae | *Casearia sylvestris* Sw. | No |
| Sapindaceae | *Cupania scrobiculata* Rich*.* | No |
| Sapindaceae | *Matayba guianensis* Aubl | Yes |
| Sapindaceae | *Paullinia rugosa* Benth. ex Radlk. | No |
| Sapotaceae | *Pouteria ramiflora* (Mart.) Radlk | Yes |
| Simaroubaceae | *Simarouba amara* Aubl | Yes |
| Turneraceae | *Turnera coerulea var. surinamensis* (Urb.) Arbo & Fernández | Yes |
| Velloziaceae | *Vellozia glauca* Pohl | Yes |
| Vochysiaceae | *Qualea grandifolia* Mart. | No |
| Vochysiaceae | *Qualea multiflora* Mart. | Yes |
| Vochysiaceae | *Qualea parviflora* Mart | Yes |
| Vochysiaceae | *Vochysia haenkeana* Mart | Yes |
| Ximeniaceae | *Ximenia americana* L. | No |
